# Protein N-terminal acetylation: NAT 2007–2008 Symposia

**DOI:** 10.1186/1753-6561-3-S6-S1

**Published:** 2009-08-04

**Authors:** Thomas Arnesen

**Affiliations:** 1 Department of Molecular Biology, University of Bergen, N-5020 Bergen, Norway; 2 Department of Surgical Sciences, University of Bergen, N-5020 Bergen, Norway; 3 Department of Surgery, Haukeland University Hospital, N-5021 Bergen, Norway

## Abstract

Protein N-terminal acetylation is a very common modification, but has during the past decades received relatively little attention. In order to put this neglected field back on the scientific map, we have in May 2007 and September 2008 arranged two international NAT symposia in Bergen, Norway. This supplement contains selected proceedings from these symposia reflecting the current status of the field, including an overview of protein N-terminal acetylation in yeast and humans, a novel nomenclature system for the N-terminal acetyltransferases (NATs) and methods for studying protein N-terminal acetylation *in vitro *and *in vivo*.

## Introduction

More than 50 years ago, the first N^α^-terminally acetylated protein was discovered [1]. Since then, numerous proteins carrying this modification have been reported, and it has become evident that protein N^α^-terminal acetylation is a major modification in eukaryotic cells. In the 1970s, work from Brown indicated that approximately 80% of the soluble proteins from mammalian cells were N^α^-terminally acetylated [2,3]. In the same period, the basic concepts of N^α^-terminal acetylation were revealed. Strous and Bloemendal demonstrated that this type of acetylation was a co-translational phenomenon, occurring on nascent polypeptide chains. N^α^-terminal acetylation occurred in an *in vitro *cell free system, when about 25–50 amino acid residues protruded from the ribosome [4,5]. In parallel, Pestana and Pitot presented data supporting the same model [6,7]. Their results strongly indicated that this reaction was catalyzed by enzyme(s) physically associated to the ribosomes and furthermore suggested that N-terminal acetylation occurs on polypeptides 40–70 amino acid residues in length.

The actual identification of the N-terminal acetyltransferases (NATs) came many years later. The yeast NatA complex, a major NAT composed of the catalytic subunit Naa10p (Ard1p) and the auxiliary subunit Naa15p (Nat1p), was identified in 1989 by the joint efforts of Sternglanz, Sherman, Grunstein and coworkers [8], and further as a physical complex in 1992 by Park and Szostak [9]. Then, Naa30p (Mak3p) [10] and Naa20p (Nat3p) [11] were identified as additional NATs with specific substrate specificities. In 1999, Sherman, Blomberg and coworkers introduced the NAT type classification based on the ability of the different NATs to acetylate different substrates: NatA (Naa10p-Naa15p), NatB (Naa20p) and NatC (Naa30p) [11]. Later, the auxiliary subunits of the NatB (Naa25p/Mdm20p) and NatC (Naa35p/Mak10p and Naa38p/Mak31p) complexes were identified by Polevoda and Sherman [12,13] and by Singer and Shaw (NatB) [14]. In 2003, an additional enzyme was discovered, Naa40p (Nat4p), making a new NAT type: NatD [15]. The same year, Rospert and co-workers provided a mechanistic insight of the NatA complex, revealing that the Naa15p subunit anchors the NatA complex to the ribosome and the nascent polypeptide, and that a potential fifth NAT, Naa50p (Nat5p), is physically associated with Naa10p and Naa15p [16]. NATs from higher eukaryotes have recently been identified [17,18] and between 2005 and 2009, all major human N-terminal acetyltransferases, NatA, NatB and NatC, were characterized [19-23]. Finally, large scale targeted proteomics has now made it possible to quantitatively analyse protein N-terminal acetylation on hundreds of unique proteins from a biological sample. Work by Arnesen and Van Damme *et al*., has revealed the conservation of substrate specificity of the NatA complex from yeast to humans, and also provided more accurate determinations of N-terminal acetylation patterns [24].

Despite some very good examples describing the importance of N-terminal acetylation at the substrate level [10,13,14,25-29], the overall importance of N-terminal acetylation remains unknown and represent an important future challenge.

Furthermore, in higher eukaryotes there might be more NATs to be discovered and there are several distinct substrate types that are acetylated by so far unknown NATs.

## Summary of the supplement

The NAT field has only had a few dedicated groups primarily focusing on N-terminal acetylation and NATs, and there is currently no scientific community specifically focusing on this field, despite the fact that it is one of the most common protein modifications, and knockout/knockdown phenotypes of NATs suggest very important functional aspects. In order to contribute to the promotion and activation of this field, we have in 2007 and 2008 arranged two symposia in Bergen, Norway. This supplement contains selected proceedings from some of the symposia participants.

An issue of importance, and discussed at these meetings, is the nomenclature of the NAT enzymes. For many reasons, including significant confusion from the non specialist scientists, all participants agreed that a novel nomenclature system for the NATs should be developed by the Sherman lab. After a round of approval among scientists in the field around the world, the new nomenclature is presented here by Polevoda *et al*. [30].

The human NATs have recently been discovered and several presentations from the group of Varhaug and Lillehaug were made at the symposia. Here, Gromyko and Starheim *et al*. have made a review of the human NATs, composition and biological significance to summarize the current knowledge of the human NATs both from their own group, but also recent results from several other research groups [31].

The next paper by Ametzazurra *et al*., describes the human NatB, which along with two previous papers [22,32] identifies this human complex composed of the catalytic subunit hNaa20p (hNat3) and the auxiliary subunit hNaa25p (hMdm20). The importance of the hNatB complex is stressed by the significant phenotypes associated with its knockdown [19].

In order to study N-terminal acetylation, it is of vital importance to have appropriate methodology available. Evjenth *et al*. describes a reliable assay for the determination of *in vitro *N-terminal acetylation, including substrate specificity and enzymatic parameters [33].

In the final paper, Van Damme *et al*. present a robust and sensitive method for assessing the N-terminal acetylation status of proteins *in vivo *[34]. The method is based on *in vitro *modifications of the protein N-termini, Cofractional Diagonal Chromatography (COFRADIC) [35] and finally Mass Spectrometry analysis. The proof of principle was very recently demonstrated in a large scale analysis of more than 1000 unique yeast and human proteins [24]. Clearly, this and similar methodologies will be crucial for the in depth studies that will be pursued in the next years.

## Competing interests

The author declares that he has  no competing interests.
